# Travelling at a slug’s pace: possible invertebrate vectors of *Caenorhabditis* nematodes

**DOI:** 10.1186/s12898-015-0050-z

**Published:** 2015-07-13

**Authors:** Carola Petersen, Ruben Joseph Hermann, Mike-Christoph Barg, Rebecca Schalkowski, Philipp Dirksen, Camilo Barbosa, Hinrich Schulenburg

**Affiliations:** Department of Evolutionary Ecology and Genetics, Zoological Institute Christian-Albrechts University, Am Botanischen Garten 1-9, 24118 Kiel, Germany

**Keywords:** *Caenorhabditis elegans*, *Caenorhabditis remanei*, Phoresy, Commensalism, Parasitism, *Arion*, Vector-mediated migration, Ephemeral habitats

## Abstract

**Background:**

How do very small animals with limited long-distance dispersal abilities move between locations, especially if they prefer ephemeral micro-habitats that are only available for short periods of time? The free-living model nematode *Caenorhabditis elegans* and several congeneric taxa appear to be common in such short-lived environments, for example decomposing fruits or other rotting plant material. Dispersal is usually assumed to depend on animal vectors, yet all current data is based on only a limited number of studies. In our project we performed three comprehensive field surveys on possible invertebrate vectors in North German locations containing populations of *C. elegans* and two related species, especially *C. remanei*, and combined these screens with an experimental analysis of persistence in one of the vector taxa.

**Results:**

Our field survey revealed that *Caenorhabditis* nematodes are commonly found in slugs, isopods, and chilopods, but are not present in the remaining taxonomic groups examined. Surprisingly, the nematodes were frequently isolated from the intestines of slugs, even if slugs were not collected in close association with suitable substrates for *Caenorhabditis* proliferation. This suggests that the nematodes are able to enter the slug intestines and persist for certain periods of time. Our experimental analysis confirmed the ability of *C. elegans* to invade slug intestines and subsequently be excreted alive with the slug feces, although only for short time periods under laboratory conditions.

**Conclusions:**

We conclude that three invertebrate taxonomic groups represent potential vectors of *Caenorhabditis* nematodes. The nematodes appear to have evolved specific adaptations to enter and persist in the harsh environment of slug intestines, possibly indicating first steps towards a parasitic life-style.

**Electronic supplementary material:**

The online version of this article (doi:10.1186/s12898-015-0050-z) contains supplementary material, which is available to authorized users.

## Background

The laboratory model system *Caenorhabditis elegans* is used in many biological disciplines, however, information on its natural life history is still scarce. A more natural context is needed to enhance our understanding of gene function, especially for those genes that are only relevant for worm life-history in the field [1]. *C. elegans* has been found frequently in ephemeral environments like rotting fruits or decomposing plant material [2–6]. These environments lack continuity because abiotic (e.g. temperature) and biotic factors (e.g. food availability) often fluctuate. Because of these fluctuations the worm seems to face a high level of unpredictability in nature, including periods with highly unfavorable conditions (e.g., high temperatures, absence of food microbes, or the presence of pathogenic bacteria), which it can escape in space, time, or a combination thereof. Dauer larvae formation represents a likely strategy for an escape in time and is very well studied under laboratory conditions [7–9]. In contrast, we currently have very little information about escape in space, especially as *C. elegans* seems unlikely to possess the necessary mobility itself considering its small size and sensitivity to desiccation [10]. *C. elegans* shares its habitats with various invertebrates and even seems to be closely associated with some of the species. These associations are commonly assumed to be part of a dispersal strategy to avoid harsh environmental conditions [11]. Interestingly, escape in space seems to be connected to escape in time, because dauer larvae are often found in association with diverse invertebrates, particularly snails, slugs, and isopods [2–4, 11–16]. The characteristic waving behavior of dauer larvae may therefore represent an adaptation to nematode-invertebrate association [11].

It is further conceivable that *C. elegans* exhibits other types of interactions with invertebrates, including necromeny and parasitism, as reported for other nematode species [11, 14, 15]. Particularly slugs show a large variety of associated nematodes which are found attached to the body or also proliferating internally [14, 17]. *C. elegans* and other *Caenorhabditis* species have been found occasionally inside of slugs [14, 17–19]. It is currently unknown whether this type of association is common or may represent an escape strategy in space with immediate access to a novel source of food, such as bacteria present in the slug’s intestines.

Here, we present our results on quantitative analysis of a wide range of invertebrates over a time span of three years to characterize their association with *Caenorhabditis* species. An initial screen focused on slugs and isopods as they are known to associate with *Caenorhabditis*. Sampling of a broader range of invertebrates subsequently aimed at identifying novel associations. These two screens revealed that *Caenorhabditis* nematodes are commonly found in the intestine of the slugs, especially of the genus *Arion*. A third screen aimed at validating this finding through a more detailed analysis of 544 slugs, mainly of the *Arion* genus, originating from 21 sampling sites. We complemented our findings with the help of two laboratory experiments, in which we assessed the ability of different nematode stages to invade and persist in the gut of *Arion* slugs across time.

## Methods

### Sampling sites and sampled invertebrates

We carried out three independent screens of invertebrates to reveal their association with common *Caenorhabditis* species. The samplings were carried out between July 2011 and October 2014. During the first screen between July 2011 and October 2012 a total of 23 slugs and 93 isopods were sampled from compost and rotten apples from three North German locations (Kiel, Münster, and Roxel). Isopods and slugs were collected in parallel to substrate samples, which we analyzed previously and found to harbor *C. elegans* and *C. remanei* and occasionally *C. briggsae* at all three sampling locations (for further details see our previous work [6]). In Kiel the invertebrates were collected in the botanical garden (54°20′N and 10°06′E) from three large compost heaps and additionally from a locally separated apple heap. In Münster, the invertebrates originated from a compost heap and apple trees in close vicinity on a meadow of the city’s farming museum (51°56′N and 7°36′E). In Roxel (51°57′N and 7°32′E) the invertebrates were collected in a private garden from three small compost heaps. A second independent screen was performed in the botanical garden in Kiel between July and September 2013 to include a broader spectrum of invertebrates. A total of 373 invertebrates (93 isopods, 56 flies, 51 chilopods, 41 spiders, 41 beetles, 35 slugs, 12 locusts, 10 bugs and 34 other invertebrates) were sampled exclusively from compost. A third independent screen was carried out between July and October 2014 to examine the potential of slug intestines for *Caenorhabditis* dispersal. 544 slugs were collected from 21 locations in Kiel or the close surroundings (Table 1). Additionally, 123 substrate samples (e.g. soil, grass, straw, leaves) were sampled from the same locations to assess whether the slugs picked up the worms at the corresponding sampling sites. The substrate samples were each collected in separate 50 ml Falcon tubes directly from underneath or within 10 cm distance to a slug. The sampling sites included six parks, four private gardens, five paths, four compost heaps, a forest and a meadow (Table 1, Additional files 1, 2, 3).

**Table 1:** Description of sampling sites used for slug mass sampling between July and October 2014

Code	Loc type	Description	GPS
G	Path^a^	Path “Schwarzer Weg”	54°20′58.4″N 10°06′51.4″E
J	Path^a^	Path “Schwarzer Weg”	54°21′35.0″N 10°07′10.2″E
P	Path^a^	Small path “Russee” along a brook, between a living area and a lake	54°18′08.0″N 10°05′12.8″E
W	Path^a^	Path “Melsdorf Landstrasse” close to street	54°18′59.4″N 10°02′08.9″E
BB	Path^a^	Path “Mühlenweg”	54°20′36.4″N 10°07′03.7″E
L	Park	Surroundings of the lake” Russee” shady because of trees	54°17′55.9″N 10°05′01.4″E
N	Park	Park “Moorteichwiesen” with some smaller water areas, frequently used by humans	54°18′37.7″N 10°07′15.1″E
R	Park	Park “Schützenwallpark” next to a lake, many stinging-nettles, frequently used by humans	54°19′00.7″N 10°06′47.4″E
U	Park	Flower bed in a park “Schlossgarten”	54°19′40.9″N 10°08′40.6″E
V	Park	Meadow in the old botanical garden, meadow is surrounded by trees	54°19′50.8″N 10°08′47.3″E
Z	Park	Park “Forstbaumschule”, meadows and trees	54°20′56.1″N 10°08′29.8″E
H	Garden	Private garden without compost	54°22′32.3″N 10°08′07.7″E
Q	Garden	Path “Russee” close to garden plots, close to a brook, apple and plum trees available	54°18′14.6″N 10°05′28.9″E
S	Garden	Private garden with apple trees in a village close to Kiel	54°13′13.2″N 10°03′54.1″E
Y	Garden	Natural finish gardens in a trailer park, apple trees and other fruit trees including rotting fruits	54°18′41.4″N 10°05′00.2″E
M	Compost	3 Big compost heaps in the botanical garden, different decomposing stages, partly covered by straw and pumpkins	54°20′47.0″N 10°07′03.8″E
O	Compost	Compost close to a beach volleyball cort, mainly grass, leaves and soil, approx. 15 m distance to a sports field	54°20′38.3″N 10°06′56.1″E
T	Compost	Private garden with compost, mostly grass and some kitchen garbage	54°20′30.2″N 10°05′52.0″E
X	Compost	Private garden with several compost heaps and an apple tree	54°20′25.1″N 10°07′35.7″E
I	Meadow	Meadow “Kopperpahler Au” with weed, separated by a path, partly next to a small river	54°20′46.5″N 10°05′27.9″E
K	Forest	Forest “Tiergehege Tannenberg”	54°21′53.0″N 10°07′04.0″E

*loc type* location type.

^a^A path either tarred or made out of sand, often some grass areas in close proximity, but without the big grass lawn found in parks (see Additional file 2A–E).

### Collection of invertebrates and isolation and identification of *Caenorhabditis* species

The invertebrates were collected and depending on their size placed individually in either 2 ml Eppendorf or in 50 ml Falcon tubes. Substrate samples were collected in plastic bags or 50 ml Falcon tubes. All invertebrates and substrates were processed within 24 h after sampling. The invertebrates were killed with a scalpel and placed individually on a peptone free medium (PFM) agar plate [20]. A spot of *Escherichia coli* OP50 was used to attract worms. Approximately 5 g of a substrate sample was placed around an OP50 spot on separate plates. Throughout the second sampling screen the slugs were analyzed in more detail. During our sampling we focused on the slug family Arionidae, which was the most frequent family to be found. The slugs were killed by cutting off the head with a scalpel. The intestines were extracted, and the slug body separated in four equally sized parts (from anterior to posterior end), in order to obtain a first approximate indication of the slug body region which contains the associated *Caenorhabditis* nematodes. Each part was analyzed for the presence of *Caenorhabditis* nematodes [20] on an individual plate. The third sampling of 2014 focused exclusively on the slug intestine. All plates were checked for nematodes within 5 h after placing the sample on the plate and again after approximately 24 and 48 h. Worms that showed characteristics of *Caenorhabditis* [20] were isolated and placed individually on 6 cm PFM plates with an OP50 spot. Occasionally occurring males where placed together with a female or hermaphrodite from the same sample. The isolated worms were left for 5–7 days at room temperature and DNA was isolated from worms that produced offspring [6]. During the sampling of various invertebrates we focused on the identification of the most common *Caenorhabditis* species found in Northern Germany. *Caenorhabditis* species were characterized following previously established and commonly used *Caenorhabditis* sampling approaches [4–6, 16, 20], based on three criteria: (1) morphological features characteristic for *Caenorhabditis* [20], (2) the production of offspring from single individuals, which is at least indicative of one of the self-fertilizing hermaphrodite taxa; and (3) a positive result in diagnostic species-specific PCRs. For identification of *C. elegans* the two primer pairs nlp30-F and nlp30-R, targeting a variable part of the immunity gene *nlp*-*30* [6], and zeel/peel-left-F and zeel/peel-left-R, targeting the *zeel*-*1*/*peel*-*1* compatibility locus [21], were used. *C. remanei* was identified using the primer pair Cre-ITS2-F1 and Cre-ITS2-R4, targeting the ribosomal internal transcribed spacer 2 (ITS2) region [6]. During the mass sampling of slugs, the primers Cbriggsae-F and Cbriggsae-R, targeting the *glp*-*1* gene, were additionally used for identification of *C. briggsae* [20]. All primer pairs have been established to be diagnostic for the indicated species [6, 17].

### Experimental analysis of the ability of *C. elegans* to invade and persist in slug intestines

To test *C. elegans’* ability to enter and persist in the slug intestine, we performed two laboratory-based experiments. In the first experiment, slugs were exposed to different stages of red fluorescent *C. elegans*, followed by microscopic analysis of dissected slugs. The slugs were freshly collected from nature. *C. elegans* of the frIs7 transgenic strain containing the p*nlp*-*29*::GFP (GFP, green fluorescent protein) and p*col*-*12*::dsRed reporters were used [22]. The p*col*-*12*::dsRed red fluorescent reporter is expressed constitutively in the epidermis, starting from the late first larval stage (L1) onwards, thus allowing identification of experimental worms in dissected intestines and thus their distinction from worms already associated with the freshly collected slugs. Approximately 15,000 synchronized *C. elegans* at either first larval (L1), fourth larval (L4), adult, or dauer larva stage were distributed on top of 25 g flower soil which was moistened with approximately 6 ml of water, a piece of cucumber and a piece of salad in a 500 ml plastic box. Each box contained only one specific stage of synchronized *C. elegans*. One slug was added to each box and boxes were closed with small meshed net to allow aeration and moistening, however preventing the escape of the slugs. Boxes without worms were used as controls. To test for worm invasion and persistence, the slugs were dissected and their intestines assessed for the presence of fluorescent worms after 24, 48 h or 6 days. At each of these time points, the intestine and the rest of the body of the killed slugs were placed separately on PFM plates with an *E. coli* OP50 spot to attract worms. For the 48 h treatment, slugs were transferred after 24 h and again after 30 h post initial exposure to a new box with fresh food and damp paper towel instead of soil, in order to separate them from fluorescent worms in the soil environment. In particular, the paper towel limits nematode survival and proliferation outside of the slug, thus minimizing the likelihood of repeated *C. elegans*-uptake by the slugs. For the 6 day-treatment, slugs were similarly transferred every 24 h to a new box with damp paper towel and fresh food. The dissected intestines and body remainder were analyzed for presence of worms 24–30 h after placing them on the PFM plate. Worm abundance was scored in five categories: no worms (category 0), 1–10 worms (category 1), 11–30 worms (category 2), 31–50 worms (category 3) and more than 50 worms (category 4). Scoring was performed without knowledge of the *C. elegans* stage that was initially added to the slug, in order to avoid observer bias. For this experiment, we analyzed a total of 31 slugs after 24 h (6 slugs for the dauer larvae treatment, 9 for the L1, 6 for the L4, 5 for the adult, and 5 for the no-worms control treatment); 25 slugs after 48 h (7 slugs for the dauer larvae treatment, 5 for the L1, 6 for the L4, 5 for the adult, and 2 for the no-worms control treatment); and 28 slugs after 6 days (9 slugs for the dauer larvae treatment, 6 for the L1, 6 for the L4, 3 for the adult, and 4 for the no-worms control treatment).

In a separate second experiment, we analyzed slug feces to assess whether *C. elegans* is able to survive the entire passage through the digestive system. A total of nine slugs were included in this experiment. Of these, two served as negative controls, which were not exposed to the fluorescent *C. elegans*. The remaining seven slugs were each exposed to approximately 15,000 fluorescent adult worms for 3 h. The worms were added first in 2 ml M9 buffer (42 mM Na2HPO_4_, 22 mM KH_2_PO_4_, 86 mM NaCl, and 1 mM MgSO_4_·7H_2_O) to 25 g wet flower soil, a piece of cucumber and salad in 500 ml plastic boxes. Slugs were placed in the box immediately after transferring the worms. The slugs were transferred to 1,000 ml boxes with fresh food but without soil in regular intervals to reduce the likelihood of worm survival outside of slugs. Within the first 12 h the feces were collected hourly to avoid secondary colonization by nematodes. Slugs were left unobserved overnight (9 h; total of 21 h after start) and feces collected every 3 h in the following 15 h (total of 36 h after start). After another 12 h (total of 48 h after start) the last feces were collected and slugs killed, resulting in a total of seven time points, for which feces were analyzed. The slug intestine was analyzed for the presence of remaining *C. elegans*. Each of the droppings was transferred to a separate 6 cm PFM plate with an OP50 spot and analyzed for the presence of fluorescent worms after 24 h.

### DNA barcoding analysis of *Arion* slug species

Species identity of a representative subset of the slugs collected in the third field screen and of the slugs used in the first experiment was characterized using an established DNA barcoding approach, based on DNA sequencing of a polymerase chain reaction (PCR)-amplified fragment of the mitochondrial cytochrome c oxidase subunit I gene (COI) [23]. DNA was extracted from intestinal slug tissue frozen at −20°C directly after the slug was killed. Approximately 25 mg of tissue was processed following the standard protocol of a DNeasy Blood & Tissue Kit (QIAGEN, Hilden, Germany). A 710 bp COI fragment was amplified using the universal primer pair LCO1490 and HCO2198 [23]. PCR was performed in 30 µl reaction volume, containing 1 µl isolated DNA, 1 unit of Taq polymerase and otherwise following polymerase manufacturer’s instructions (Promega, Madison, USA). PCR cycling consisted of 95°C for 2 min, followed by 35 cycles of 95°C for 1 min, 40°C for 1 min and 72°C for 1.5 min, and a final extension step at 72°C for 7 min. The PCR product was directly subjected to Sanger sequencing in both directions with the PCR primers at the Sequencing facility of the Institute of Clinical Molecular Biology, Kiel University, Germany. For each sample, the resulting corresponding two sequences were aligned and a consensus sequence was produced for the overlapping part, yielding a mean fragment length of 594.8 (±2.52 standard error of the mean, SEM). This fragment was subjected to a BLAST comparison with the public NCBI Nucleotide collection (nr/nt) database [24]. We recorded species designations of the first three most similar sequences, which in all cases showed a similarity of more than 99.1% (average BLAST bitscore of 1095 ± 4.31 SEM).

### Statistics

The current study explores the association of *Caenorhabditis* with various invertebrates. Three types of statistical tests were applied with caution to each invertebrate group or body part separately, in order to assess the overall variation in species prevalence or nematode occurrence in a certain group or part. We used Fisher’s exact test for pairwise comparisons of the number of independent invertebrate individuals containing either of the different *Caenorhabditis* species. The comparison was performed across the entire sampling period and for each invertebrate group separately. Fisher’s exact test was also used for comparison of nematode abundance in different slug parts. The first experiment on the abundance of nematode stages in slug intestines and remainder was compared using ANOVA and posthoc pairwise comparisons with Tukey’s HSD test. The second experiment on the amount of worms in feces across time was also compared with an ANOVA. All statistical tests were performed with the program R version 3.1.1. For each of the analyses, multiple testing was accounted for by adjusting the significance level using the false discovery rate (FDR; [25]). Graphs were produced with R version 3.1.1 and Inkscape version 0.48.

## Results

### First screen: both *Caenorhabditis* species are associated with isopods and slugs

93 isopods (69 from compost, 24 from rotten apples) and 23 slugs (21 from compost, two from rotten apples) were collected from three North German locations between July 2011 and October 2012 and analyzed for the presence of *Caenorhabditis* species. Our aim was to obtain a first approximate idea of the invertebrate groups, which harbor the *Caenorhabditis* nematodes in Northern Germany. Therefore, we did not determine exact species identities of all collected isopods and slugs. We nevertheless noted that isopods mainly included three of the species that are abundant in Northern Germany, namely *Porcellio scaber*, *Oniscus asellus*, and *Armadillidium vulgare*, while almost all slugs belonged to the genus *Arion*. In addition, our previous work showed that substrate samples (i.e., compost material and/or rotten apples) from all three sites can harbor *C. elegans, C. remanei* and occasionally *C. briggsae* [6]. Since *C. elegans* and *C. remanei* were the dominant species in the substrates we focused on these two species. *C. elegans* and *C. remanei* were both found in association with isopods and *C. elegans* additionally with slugs (Additional file 4). *C. elegans* was isolated from eight isopods (8.6% of all isopods) and eight slugs (34.8% of all slugs). One isopod from compost carried both *Caenorhabditis* species simultaneously. *C. remanei* was found on 14 isopods (15.1% of all isopods), but was not associated with slugs during this screen. Compost isopods carried *C. elegans* (11.6%; 8 of 69 isopods) and *C. remanei* (10.1%; 7 of 69 isopods), whereas apple isopods never carried *C. elegans,* but only *C. remanei* (29.2%; 7 of 24 isopods). Consistent with this finding the apples from which the isopods were collected were not found to contain any *C. elegans* [6].

### Second screen: analysis of associations with a large variety of invertebrate taxa

**Figure 1:**
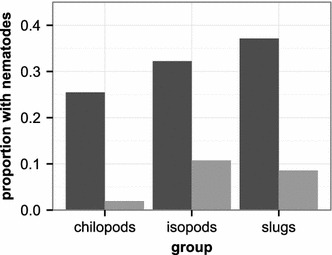
Proportion of chilopods, isopods and slugs found in association with *C. elegans* and *C. remanei* during the second screen between July and September 2013. Neither *C. elegans* (*dark grey*) nor *C. remanei* (*light grey*) have been found associated with other invertebrates. The overall occurrence of *C. elegans* differed from that of *C. remanei*, yet each species was isolated in similar relative frequencies from the three invertebrate groups (*C. elegans* was found in 13 out of 35 assayed slugs, 30 out of 93 isopods, and 13 out of 51 chilopods; *C. remanei* was isolated from 3 out of 35 slugs, 10 out of 93 isopods, and 1 out of 51 chilopods; Additional file 5).

373 invertebrate specimens from various taxonomic groups were analyzed for the presence of the two *Caenorhabditis* species between July and September 2013*. C. elegans* and *C. remanei* were found in association with 15 or 3.8% of the sampled invertebrates, respectively, all of them from three invertebrate groups: slugs, isopods, and chilopods (Figure 1; Additional file 5). *C. elegans* and *C. remanei* differed significantly in abundance on the three invertebrate groups (Fisher’s exact test for a r x c contingency table, total n = 179, *P* < 0.001; FDR-adjusted for multiple testing at α < 0.05): 13 of 35 slugs (37.1%), 30 of 93 isopods (32.3%) and 13 of 51 chilopods (25.5%) carried *C. elegans*, whereas *C. remanei* was isolated from three of 35 slugs (8.6%), 10 of 93 isopods (10.8%) and one of 51 chilopods (2%). As for the first screen, we focused on only the broad taxonomic invertebrate groups and did not characterize species identities. We noted again that most isopods belonged to *P. scaber*, *O. asellus*, and *A. vulgare*, while almost all slugs were from the genus *Arion*. Separate analysis of the invertebrate groups showed no significant difference in *C. elegans* and *C. remanei* occurrence on either slugs, isopods, or chilopods (in all cases, Fisher exact test for 2 × 2 tables, *P* > 0.1). *C. elegans* and *C. remanei* co-occurred in one slug, three isopods and one chilopod. 56 flies, 41 spiders, 41 beetles, 12 locusts, 10 bugs and 34 other invertebrates did not carry any *C. elegans* or *C. remanei*.

**Figure 2:**
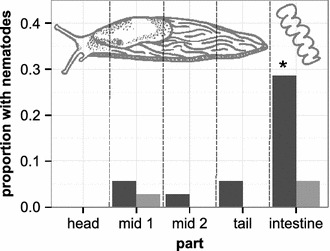
Proportion of different slug body sections and intestine associated with either *C. elegans* or *C. remanei* during the second screen between July and September 2013. *C. elegans* (*dark grey*) and *C. remanei* (*light grey*) proportions were calculated in relation to the total number of 35 slugs analyzed for this screen. The only value with significant variation to all others is indicated by an *asterisk*.

The collected slugs were additionally dissected to assess exact localization of the nematodes. Head, two middle parts (mid 1 and mid 2), tail and intestine were analyzed separately and variation in worm prevalence among slug parts was tested with Fisher’s exact test for r x c contingency tables (Figure 2, including the total of 35 slugs for both *Caenorhabditis* species). Significantly more *C. elegans* were found in the intestine compared to the head region (Fisher’s exact test, n = 35, *P* = 0.008; FDR-adjusted for multiple testing at α < 0.05) and the second middle part (*P* = 0.027) and numbers in the intestine tended to be different to the first middle part (*P* = 0.052) and the tail (*P* = 0.052). The abundance of *C. elegans* was not significantly different between head, the two middle parts and tail (in all cases, *P* > 0.1). There were no significant differences in *C. remanei* occurrence between slug parts (in all cases, *P* > 0.1).

### Third screen: comprehensive analysis of *Caenorhabditis* species prevalence in slug intestines

**Figure 3:**
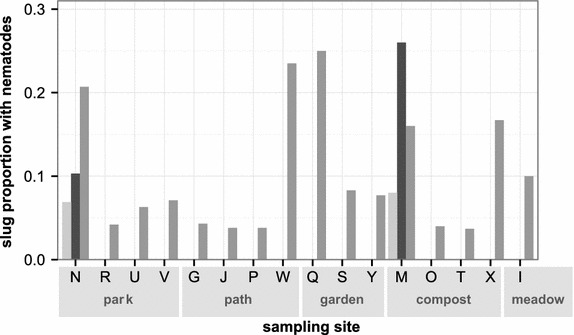
Occurrence of *C. elegans* (*dark grey*), *C. remanei* (*grey*) and *C. briggsae* (*light grey*) in slug intestines during the third screen between July and October 2014. *Letters* indicate the different sampling sites and are grouped by location type (see also Table 1). Slug intestines from five sampling sites did not harbor any *Caenorhabditis* (sites H, K, L, Z and BB; data not shown). The indicated proportions are always calculated in relation to the total number of slug intestines assayed at the corresponding sampling site (Table 2; Additional file 6).

We characterized the intestines of a total of 544 slugs (almost all of the genus *Arion* and one *Limax maximus*; see also below) between July and October 2014. We found nematodes of three *Caenorhabditis* species in 54 of these (9.9%), originating from 16 of 21 sampling sites (76.2%; Figure 3; Table 2; Additional file 6). The sampling sites were grouped in several broad categories of location types (i.e., park, garden, compost, forest, etc.; see Table 1 and Additional files 1, 2) with general differences in structure and habitat properties, in order to assess to what extent such location differences may influence the occurrence of *Caenorhabditis*-containing slugs. *C. remanei* was found in 45 (8.5% of 544 slugs; Table 2), *C. elegans* in 15 (2.8%) and *C. briggsae* in 6 slugs (1.1%). *C. remanei* thus occurred significantly more often than *C. elegans* (Fisher’s exact test for a 2 × 2 table, n = 544, *P* < 0.001; FDR-adjusted for multiple testing at α < 0.05) and *C. briggsae* (*P* < 0.001), while *C. elegans* tended to occur more often than *C. briggsae* (*P* = 0.05). Variation in *C. remanei* prevalence could be explained by the sampling site (ANOVA, *P* = 0.002) but not the different location types (see Table 1) or the interaction between these two (in both cases, *P* > 0.1). Sampling sites (ANOVA, *P* < 0.001) but not location types or the interaction between both (*P* > 0.1) accounts for variation in *C. elegans* occurrence. Neither the location nor the location type nor the interaction between the two factors explained the variation in *C. briggsae* occurrence (in all cases, *P* > 0.1).

**Table 2:** Slugs and substrates associated with *Caenorhabditis* nematodes during the third screen in 2014

Site	Loc type	Total^a^		With *CR* ^b^		With *CE* ^b^		With *CB* ^b^	
		Slugs	Sub	Slugs	Sub	Slugs	Sub	Slugs	Sub
L	Park	30	4	0 (0)	0 (0)	0 (0)	0 (0)	0 (0)	0 (0)
N	Park	29	8	6 (0.21)	0 (0)	3 (0.1)	0 (0)	2 (0.07)	1 (0.13)
R	Park	24	5	1 (0.04)	0 (0)	0 (0)	0 (0)	0 (0)	0 (0)
U	Park	32	6	2 (0.06)	0 (0)	0 (0)	0 (0)	0 (0)	0 (0)
V	Park	28	6	2 (0.07)	0 (0)	0 (0)	0 (0)	0 (0)	0 (0)
Z	Park	23	5	0 (0)	0 (0)	0 (0)	0 (0)	0 (0)	0 (0)
BB	Path	13	2	0 (0)	0 (0)	0 (0)	0 (0)	0 (0)	0 (0)
G	Path	23	5	1 (0.04)	0 (0)	0 (0)	0 (0)	0 (0)	0 (0)
J	Path	26	7	1 (0.04)	0 (0)	0 (0)	0 (0)	0 (0)	0 (0)
P	Path	26	4	1 (0.04)	0 (0)	0 (0)	0 (0)	0 (0)	0 (0)
W	Path	17	3	4 (0.24)	0 (0)	0 (0)	0 (0)	0 (0)	0 (0)
H	Garden	28	8	0 (0)	0 (0)	0 (0)	0 (0)	0 (0)	0 (0)
S	Garden	24	5	2 (0.08)	0 (0)	0 (0)	0 (0)	0 (0)	0 (0)
Q	Garden	24	4	6 (0.25)	0 (0)	0 (0)	0 (0)	0 (0)	0 (0)
Y	Garden	26	11	3 (0.12)	2 (0.18)	0 (0)	0 (0)	0 (0)	0 (0)
M	Compost	50	10	8 (0.16)	0 (0)	12 (0.24)	3 (0.3)	4 (0.08)	1 (0.02)
O	Compost	25	7	1 (0.04)	0 (0)	0 (0)	0 (0)	0 (0)	0 (0)
T	Compost	27	6	1 (0.04)	1 (0.17)	0 (0)	0 (0)	0 (0)	0 (0)
X	Compost	18	6	3 (0.17)	1 (0.17)	0 (0)	0 (0)	0 (0)	0 (0)
I	Meadow	30	6	3 (0.1)	0 (0)	0 (0)	0 (0)	0 (0)	0 (0)
K	Forest	21	5	0 (0)	0 (0)	0 (0)	0 (0)	0 (0)	0 (0)

*CR* *C. remanei*, *CE* *C. elegans*, *CB* *C. briggsae*, *loc type* location type, *sub* substrate sample.

^a^Total number of independent slugs or substrate samples.

^b^Number and proportion (in brackets) of independent slugs or substrate samples that contained the respective *Caenorhabditis* species per site.

*C. elegans* and *C. remanei* co-occurred in five slugs, four from compost (sampling site M) and one from a park (sampling site N). *C. elegans* and *C. briggsae* were isolated together from one slug and one substrate sample from compost (sampling site M). *C. elegans*, *C. remanei* and *C. briggsae* co-occurred in three slug intestines, one sampled from compost and two in a park (sampling site M or N, respectively). We also scored the stage of the *Caenorhabditis* that were isolated within 5 h after placing the intestine on plate. Three out of twelve isolated *C. remanei* were adults originating from sampling site G and Q (2×), one was a third instar larvae (L3; placed together with a male; sampling site G) and all other eight isolates were dauer larvae. We only isolated a single *C. elegans* and a single *C. briggsae* at this early time point and both were dauer larvae. Of the 123 additionally collected substrate samples (Additional file 6), eight (6.5%) contained *Caenorhabditis* nematodes. *C. remanei* was found in four substrates, two originating from compost (sampling site T and X) and two from a garden with rotten apples (sampling site Y). *C. elegans* was found in three compost samples (sampling site M) and *C. briggsae* occurred in two substrates (compost M and park N).

We used a DNA barcoding approach on a subset of the collected slugs to obtain a better understanding of the exact species, which harbored the different *Caenorhabditis* species. For this analysis, a total of 252 slugs was characterized (Additional files 6, 7). 194 of these belong to *Arion lusitanicus* (77.0% of the total of 252), 55 to uncharacterized *Arion* species (21.8%), and one each to *A. rufus* and *A. subfuscus* (0.4% in each case). We also confirmed identity of *L. maximus* with this approach. 23 individuals of *A. lusitanicus* harbored *Caenorhabditis* nematodes (11.9% of the total number of analyzed *A. lusitanicus*). Of these, 15 contained *C. remanei* (7.7% of all 194 tested *A. lusitanicus*), 4 *C. elegans* (2.1%), and 4 *C. briggsae* (2.1%). 16 individuals of uncharacterized *Arion* species had *Caenorhabditis* nematodes (29.1% of the 55 examined individuals of this group), twelve with *C. remanei* (21.8%) and four with *C. elegans* (7.3%). The other two identified *Arion* species did not harbor any *Caenorhabditis* worms, whereas *L. maximus* was associated with both *C. elegans* and *C. remanei* (Additional file 7). Taken together, the two most common *Arion* taxa (*A. lusitanicus* and uncharacterized *Arion* species) were most often associated with *Caenorhabditis* nematodes, especially with the most frequent nematode species of this screen, *C. remanei*.

### Slug experiment: various *C. elegans* stages can enter and survive the slug intestine

**Figure 4:**
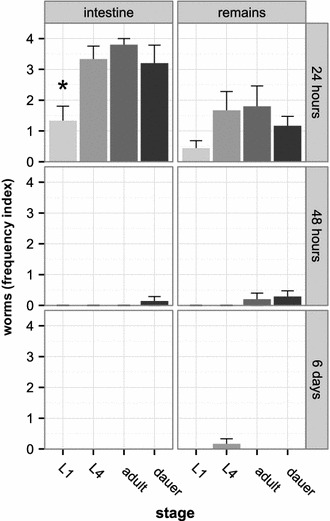
Different *C. elegans* stages were found in slug intestines and body remainder. The experiment was based on characterization of a total of 84 slugs. Of these, 31 were analyzed after 24 h (*top row*; 6 slugs for the dauer larvae treatment, 9 for the L1, 6 for the L4, 5 for the adult, and 5 for the no-worms control treatment); 25 slugs after 48 h (*middle row*; 7 slugs for the dauer larvae treatment, 5 for the L1, 6 for the L4, 5 for the adult, and 2 for the no-worms control treatment); and 28 slugs after 6 days (*bottom row*; 9 slugs for the dauer larvae treatment, 6 for the L1, 6 for the L4, 3 for the adult, and 4 for the no-worms control treatment). The graph does not show results for the control treatment, because these did not contain any of the labeled *C. elegans*. For the other treatments (given along the X axis), the presence of worms was separately analyzed for slug intestines and the remainder of the body (*left* and *right columns*, respectively). Worms were counted in categories (category 0 = no worms, category 1 = 1–10 worms, category 2 = 11–30 worms, category 3 = 31–50 worms, category 4 = more than 50 worms). For illustration, we calculated a frequency index by taking the average of the ordered categories per worm stage, slug body part, and time point. The Y axes show the worm frequency indices (±standard error). *C. elegans* L1, L4, adult and dauer larva stages were able to enter the intestine of slugs within 24 h. Worms associated also with the outside of the slugs and could be found on the remains. After 48 h (24 h after separating the slugs from the worms) almost no worms were found in the intestine or on the remainder. The only value that differed significantly from all others from the same body part and time point is indicated by an *asterisk.*

We tested the ability of different *C. elegans* stages to enter and persist the intestines of slugs in two experiments. We used red fluorescent *C. elegans* to distinguish the experimental worms from nematodes that may have already been associated with the slugs, which were originally collected from nature. DNA barcoding analysis of a representative subset of slugs from the first experiment revealed that 66.7% (22 out of 33 tested; Additional file 8) belong to *A. lusitanicus* and the rest to uncharactized *Arion* species (33.3%; 11 out of 33; Additional file 8). In this first experiment, *C. elegans* was found in the intestine and on the remainder of the body after 24 h post initial exposure, but rarely after 48 h or 6 days (Figure 4). The statistical analysis was thus focused on the 24 h exposure time point (original data available from the dryad repository, doi:10.5061/dryad.9j850). At this time point, the overall number of worms was significantly higher in the intestine compared to the remainder (ANOVA, *P* < 0.001; analysis based on a total of 26 slugs). In general, all stages were found in the intestine and on the rest of the body. Moreover, the frequencies of the various *C. elegans* stages in the intestines varied significantly (ANOVA, *P* = 0.003; analysis based on the 26 slugs; Figure 4). Dauer larvae tended to be more frequent in the intestine than the remainder (Tukey HSD, *P* = 0.089), whereas no such difference was significant for the other stages (in all cases, Tukey HSD, *P* > 0.1). A pairwise comparison of the frequencies of the various *C. elegans* stages in the slug intestines at the 24 h time point additionally revealed that L1 stages were significantly less frequent than L4 (Tukey HSD, *P* = 0.019; analysis based on the 26 slugs; Figure 4), adults (Tukey HSD, *P* = 0.006) and dauer larvae (Tukey HSD, *P* = 0.044). The number of adults, L4 s, and dauer larvae from slug intestines did not differ significantly between each other (Tukey HSD, *P* > 0.1), while in the slug remainder pairwise comparison of the frequencies of the various nematode stages did not yield any significant difference (Tukey HSD, *P* > 0.1).

### Experimental analysis of feces

**Figure 5:**
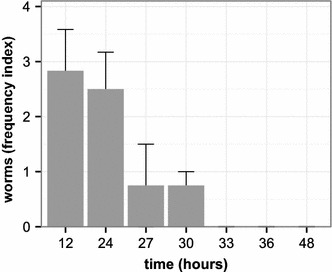
*C. elegans* adults in slug feces across time after 3 h of initial exposure to nematode cultures. The experiment was based on nine slugs. Two of these served as negative controls and were not exposed to *C. elegans*. The remaining seven slugs were exposed to adult *C. elegans*, revealing a decrease in *C. elegans* abundance in slug feces over time (X axis). Worms were counted in categories (category 0 = no worms, category 1 = 1–10 worms, category 2 = 11–30 worms, category 3 = 31–50 worms, category 4 = more than 50 worms). For illustration of our findings, we calculated a frequency index for each time point by taking the average of the category scores (which are ordered in size according to worm frequency ranges) across the studied slugs per time interval. The Y axis denotes the calculated frequency indices (±standard error). All scored worms were alive.

To test whether and for how long the nematodes are able to survive the entire passage through the digestive system of the slug we analyzed slug feces for the presence of living fluorescent nematodes (Figure 5). We found that *C. elegans* adult stages are able to enter and survive the passage, but the number of worms decreased significantly over time (ANOVA, *P* < 0.001; analysis based on seven slugs studied across seven time points; original data available from the dryad repository, doi:10.5061/dryad.9j850). Worms could no longer be recovered from the feces after 30 h. Additionally, the intestines of all slugs were dissected at the end of the experiment (48 h) and did not contain any nematode. *C. elegans* recovered from feces were generally fertile, because we repeatedly observed eggs and L1 larvae on the assay plates, onto which feces had been transferred.

## Discussion

### *Caenorhabditis* association with possible invertebrate vectors

In this study we explore the importance of possible vectors for small-sized animals that live in ephemeral habitats using the model nematode *C. elegans*. *C. elegans* is suited for such studies for three main reasons. Firstly, *C. elegans* and several congeneric taxa are common inhabitants of short-lived environments [3, 6, 11]. Secondly, field studies can be efficiently combined with laboratory experiments in these taxa, because of the ease with which this nematode can be controlled and manipulated under laboratory conditions. Thirdly and most importantly, the available toolkit for *C. elegans* functional genetic analysis can in the future be used to dissect the genes involved in interactions with invertebrate vectors. This information is currently not available for other taxa with similar life history and would similarly help to enhance our understanding of the biology of this intensively studied model taxon. Interactions with invertebrates are assumed to be an escape strategy used by the worms under unfavorable environmental conditions. In our field survey we analyzed a total of 1034 invertebrates of different taxonomic groups for the presence of common *Caenorhabditis* species and revealed that these are commonly found in slugs, isopods, and chilopods, however are absent in other invertebrate taxa. We found indications for the presence of *C. elegans* in slug intestines, in the large third screen especially the slug *A. lusitanicus*. We then exposed 93 slugs to a total of 1200000 worms of different stages in two experiments (15000 worms to each of 80 slugs plus 13 control slugs). We confirmed hereby the ability of *C. elegans* to invade and persist for a short time in slug intestines and subsequently to be excreted alive with the slug feces, possibly indicating first steps towards a parasitic life-style.

Our findings can be placed in context of our current understanding of *Caenorhabditis* ecology. Most *Caenorhabditis* species have been found in microbe-rich organic material [3, 4, 6], an environment which they often share with various invertebrates. Several invertebrate taxa including isopods, millipedes, snails, and slugs were previously reported to harbor *Caenorhabditis* nematodes [2–4, 11–17, 26]. Our comprehensive screens revealed that *C. elegans* and *C. remanei* are commonly associated with slugs, isopods and chilopods, but with none of the other invertebrate taxa studied. Humidity may be of key importance for the ability of nematodes to attach to invertebrates, consistent with our previous observation of the influence of humidity for the general occurrence of *Caenorhabditis* species in rotting plant material [6]. Most nematode stages suffer severely from dehydration if exposed to dry environments [10, 11]. Even *C. remanei* dauer larvae were previously observed to stay for up to 5 days attached to their isopod host in dry environments but abandoned the isopod immediately in a damp environment [12]. *C. elegans* and related species may take advantage of the moist micro-environment encountered in some invertebrates, especially snails and slugs, which have previously been found to harbor *Caenorhabditis* nematodes [14, 26]. These gastropod groups constantly produce mucus, for example to aid locomotion [27, 28] or to attach to substrates [29], thus providing a generally humid environment favorable for the nematodes. As discussed in more detail below, slug intestines may be even more advantageous because they provide humidity as well as potential food microbes.

Neither *C. elegans* nor *C. remanei* occurred on flies, beetles, spiders, locusts and bugs or other invertebrates although these invertebrates were collected from the same compost as the worm-containing slugs, isopods and chilopods. This suggests that initiation or maintenance of infestation in the former groups is somehow constrained. One reason may be lack of sufficient humidity. Alternative explanations may be a consequence of nematode chemosensation, choice behavior, and/or possible defenses of the invertebrate taxa. In particular, worms may be repelled by chemical defense mechanisms, used by numerous invertebrates, such as beetles [30] or harvestmen [31]. Such chemical defense compounds may repulse or prevent *C. elegans* from attaching. Recognition of a carrier invertebrate may also be species-specific. Host preference has been observed for *C. remanei* within Porcellionidae, a family of isopods [12]. It has also been found in other *Caenorhabditis* species, such as dauer larvae of *C. japonica* and their attraction towards the burrower bug *Parastrachia japonensis* [15]. Chemotactic attraction of nematodes towards invertebrate hosts is currently best described for *Pristionchus pacificus*, which is associated with scarabaeid beetles [32]. These nematodes are able to specifically detect chemical signatures of their host beetle species and then use these to navigate towards their hosts [32]. It is as yet unclear how *C. elegans* is able to specifically detect and respond to cues of invertebrate taxa in its natural environment. Analysis of such interactions may be of particular value for our understanding of the worm’s biology and could be comparatively easily achieved in the future using the established toolkit of *C. elegans* behavioral assays and functional genetic analysis methods.

### Moving at slug’s pace

Our analysis highlighted that *C. elegans* is particularly common in slug intestines when compared to the rest of the slug body, especially in the most abundant slug species *A. lusitanicus*. This finding confirms the previous reports of *C. elegans* inside of slugs collected in Africa [14] and Germany [17] and of *C. briggsae* inside of slugs from the US [18]. In these three previous studies a wide spectrum of slug-associated nematodes was explored and Ross et al. examined mainly parasitic nematodes, thus, *C. elegans* ecology was not the primary focus. In our study we analyzed 544 slug intestines from 21 sampling sites in Kiel, Germany, and were able to recover all three species, *C. remanei, C. elegans*, and *C. briggsae*. The prevalence of *C. elegans* and *C. remanei* in slug intestines varied among the two relevant screens, possibly due to random differences among years or other factors, which were not controlled. It would be interesting in the future to assess which exact factors may account for such variation. Although *Caenorhabditis* was found in slugs from 76.2% of the sampling sites, most of the directly associated substrate samples (e.g., substrate collected below or directly adjacent to the studied slugs) did not harbor any *Caenorhabditis*. These results are a possible indication that worms were picked up earlier and already transported by the slugs for some time. This strongly indicates that these worms are able to survive at least the time required to migrate from nematode containing substrates to the sampling location in the intestinal environment. In addition, to our knowledge, our study is the first to have isolated the three species, *C. elegans*, *C. remanei,* and *C. briggsae*, from the same substrate sample. In this case, the individual worm-mixture-containing slugs were collected from the compost in the botanical garden and a park in Kiel. This finding implies that all three species coexist, two or more *Caenorhabditis* species can directly interact in nature, and thus they may indeed compete for the same or at least a related ecological niche [3, 6]. Our screen was specifically focused on the nematode genus *Caenorhabditis*. We regularly noticed presence of other nematode taxa, which could represent additional competitors for the *Caenorhabditis* species and should thus be considered in future studies.

### Various *C. elegans* stages can enter and leave the slug intestine

The dauer larva stage is predominantly found in association with invertebrates [11, 12]. This stage has thus been suggested to be specifically adapted for attachment and subsequent transport by vectors, especially if worms attach to the outside of other invertebrates [11]. Interestingly, even though dauer larvae were most frequent, we occasionally also observed other stages in the slug intestines in our field survey. Thus, different stages seem to be able to enter and persist in slug intestines, possibly because slugs unintentionally take up any of the stages while feeding on rotting plant material. Alternatively, slugs ingest dauer larvae which subsequently form proliferating populations. With our experiments, we specifically tested which *C. elegans* stages are able to enter and persist in the intestines. Our results highlight that all stages are able to enter, but not with similar efficiency. L1 worms were less proficient in establishing themselves in the slug gut than the other tested stages. One possible explanation is that the L1 stage is less likely to pass the slug radula unharmed or to resist the digestive system. Nevertheless, even though various stages are able to enter slugs, our finding from the field survey of a high abundance of dauer larvae suggests that this stage is particularly favored under natural conditions to be taken up by slugs, possibly because of specific behavioral adaptations (e.g., nictation behavior [9, 11]) or because dauer larvae are common on substrates preferred by the slugs.

During the experiments, the tested *C. elegans* stages were not able to persist for much more than 1 day in the intestines, indicating an only short-term interaction with the slug. These results contrasted with the field results, where worm-containing slugs were often found in no close association with substrates suitable for *Caenorhabditis* proliferation (e.g., on sidewalks close to streets or on large grass areas without rotting plant material), which may imply that worms can travel with the slugs for more than 1 day. Nevertheless, the same outcome may also be achieved by repeated re-invasion of the slugs. Alternatively our experimental conditions differ too much from field conditions, especially as to the maintenance of the slugs. It would be of particular value in the future to quantify the exact dynamics of *C. elegans*-slug interactions under natural conditions, for example by assessing slug feces collected from distinct field locations, which either offer or lack suitable nematode substrates.

Similarly, based on our results, it is not entirely clear which type of association is formed between *C. elegans* and slugs. A purely phoretical interaction with invertebrates with weak or no effects on host fitness has previously been proposed for *C. elegans* [11]. In our study we found that the worms enter and leave the slug intestine without any obvious harm and generally being fertile, suggesting that slugs represent suitable means of transport. At the same time, the slugs survived infestation with large worm numbers without any obvious damage. Both findings support the idea of a phoretic association, but are also consistent with a commensal or even mutualistic interaction. In addition, the slug’s bacterial community may be exploited by *C. elegans* as food during the short-term inhabitation. This may be supported by our finding of occasional non-dauer larva stages in the slug intestines, possibly suggesting that the nematodes proliferate and reproduce inside the slug, Moreover, although rarely found for *Caenorhabditis* [11], a parasitic association between *C. elegans* and slugs may not yet be excluded. In fact, parasitic relationships are known for other nematodes that use slugs as intermediate and final hosts [14, 17, 18, 33, 34]. One prominent example is the commercially available strain *Phasmarhabditis hermaphrodita* [35], which actively searches for slugs and kills them through infection with its gut bacteria [36]. A distinction of these alternative interaction types requires a more detailed, long-term analysis of the *C. elegans*-slug associations under controlled conditions.

## Conclusions

*C. elegans* and *C. remanei* can be regularly found in association with diverse invertebrates in Northern Germany being more prevalent on slugs, isopods and chilopods than on other taxa including beetles, flies and spiders possibly as a consequence of carrier specificity or chemical host defense mechanisms. *Caenorhabditis* nematodes can especially be found in slug intestines in higher frequencies. The exact nature of this association is hitherto unknown. Our analysis indicates that slugs are a suitable means of transport for diverse *Caenorhabditis* species in different developmental stages and that slug intestines may provide more advantages during dispersal hinting on possible mutualistic, commensal or possibly even parasitic interactions. Therefore, detailed long-term analysis of the *C. elegans*-slug associations under controlled conditions may provide a better understanding on the nature of the interaction and the underlying dynamics.

### Availability of supporting data

The original data for the three field screens is provided in Additional files 4, 5, and 6. The original data for the two experiments are available from the Dryad Digital Repository: doi:10.5061/dryad.9j850. The COI DNA sequences for slug DNA barcoding are available from genbank under accession numbers KR867347–KR867631.

## Additional files

Additional file 1: Overview of sampling sites. The pictures show some of the 21 sampling sites, from which slugs were collected, including six parks (A-F) and four compost heaps (G, H, J, K). Picture I shows a slug on compost. The other sampling sites are shown in Additional file 3. Letters in brackets refer to the code used for the individual sampling sites (Table 1). Additional file 2: Overview of sampling sites. The pictures show some of the 21 sampling sites, from which slugs were collected, including five paths (A-E), four private gardens (F-I), one meadow (J) and one forest (K). The other sampling sites are shown in Additional file 2. Letters in brackets refer to the code used for the individual sampling sites (Table 1). Additional file 3: Map of sampling locations in Kiel. Slugs were sampled from 21 locations in Kiel and the surroundings. Different letters indicate different locations. For details see Table 1 and Additional Files 2 and 3. Location S is not shown as it is outside of Kiel. Additional file 4: Original data for the first field screen in 2011 and 2012. *C. elegans* and *C. remanei* were found in association with isopods and slugs during the first screen between July 2011 and October 2012. Additional file 5: Original data for the second field screen in Kiel in 2013. 373 invertebrates from various taxonomic groups were sampled between July and September 2013. *C. elegans* and *C. remanei* were found in association with chilopods, isopods and slugs. Additional file 6: Original data for the third field screen in Kiel, Northern Germany, in 2014. 544 slugs and 123 substrate samples were collected between July and October 2014 from 21 locations in Kiel and the surroundings. Additional file 7: Association of different slug species from the third field screen with *C. elegans*, *C. remanei* or *C. briggsae*. Slug species identity was characterized for a representative subset of the slugs from the third screen with a DNA barcoding approach using a fragment of the mitochondrial COI gene. Additional file 8: Overview of slug species used in the first laboratory experiment. Species identity was determined for a subset of slugs with a DNA barcoding approach using a fragment of the mitochondrial COI gene.

## Abbreviations

ANOVA: analysis of variance
COI: cytochrome oxidase subunit I
FDR: false discovery rate
GFP: green fluorescent protein
ITS2: internal transcribed spacer 2 of the ribosomal cistron
L1: first instar nematode larvae
L3: third instar nematode larvae
L4: fourth instar nematode larvae
M9: nematode medium (42 mM Na2HPO_4_, 22 mM KH_2_PO_4_, 86 mM NaCl, 1 mM MgSO_4_·7H_2_O)
NGM: nematode growth medium
PCR: polymerase chain reaction
PFM: peptone free nematode growth medium
SEM: standard error of the mean
