# Supplementary figures and images for: Travelling at a slug’s pace: possible invertebrate vectors of Caenorhabditis nematodes
Source: BMC Ecol. 2015 Jul 13;15:19. doi: 10.1186/s12898-015-0050-z (PMC4501285; doi:10.1186/s12898-015-0050-z)

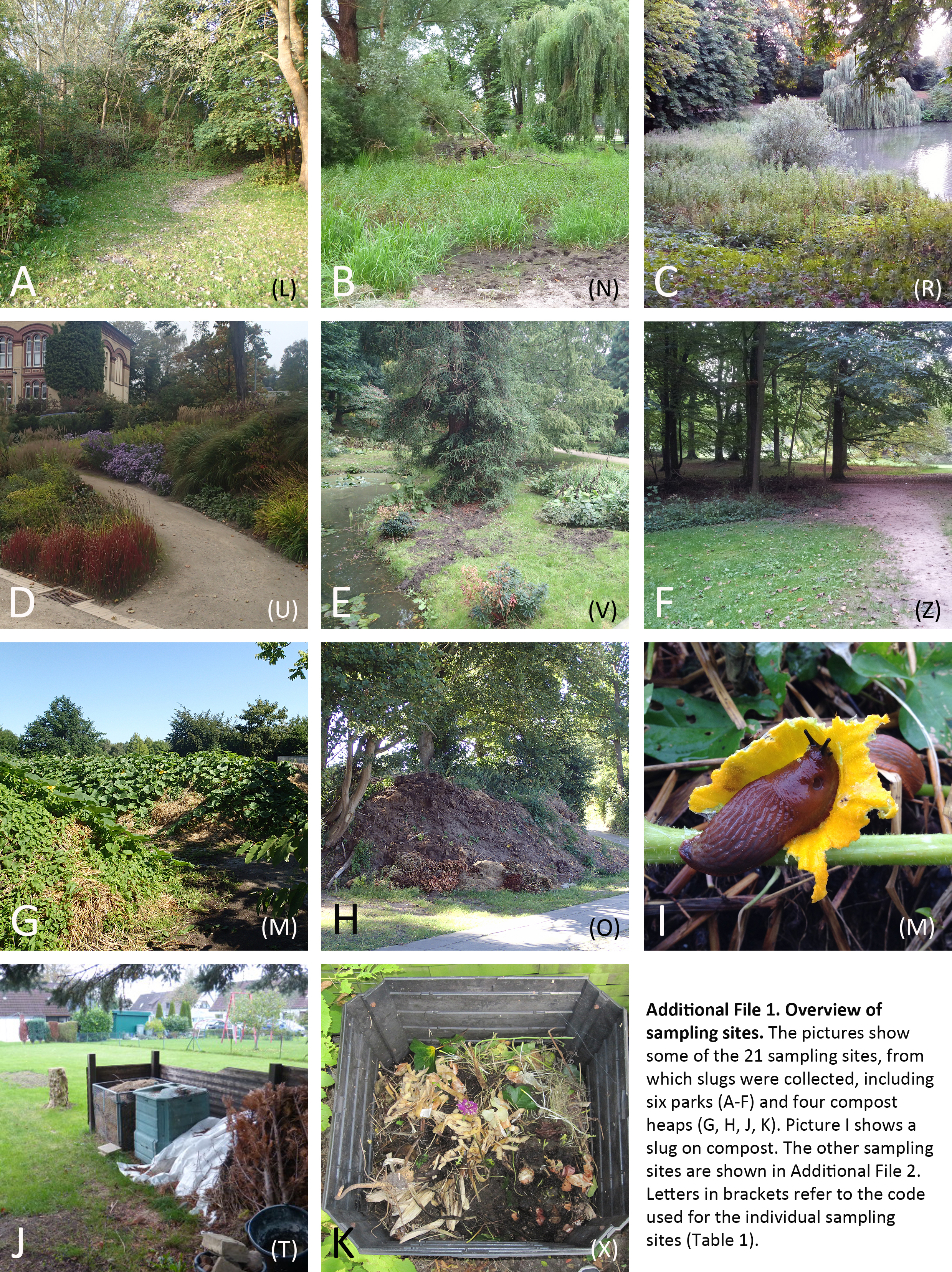

Supplement: Additional file 1: — Overview of sampling sites. The pictures show some of the 21 sampling sites, from which slugs were collected, including six parks (A-F) and four compost heaps (G, H, J, K). Picture I shows a slug on compost. The other sampling sites are shown in Additional file 3. Letters in brackets refer to the code used for the individual sampling sites (Table 1). [file 12898_2015_50_MOESM1_ESM.jpg]

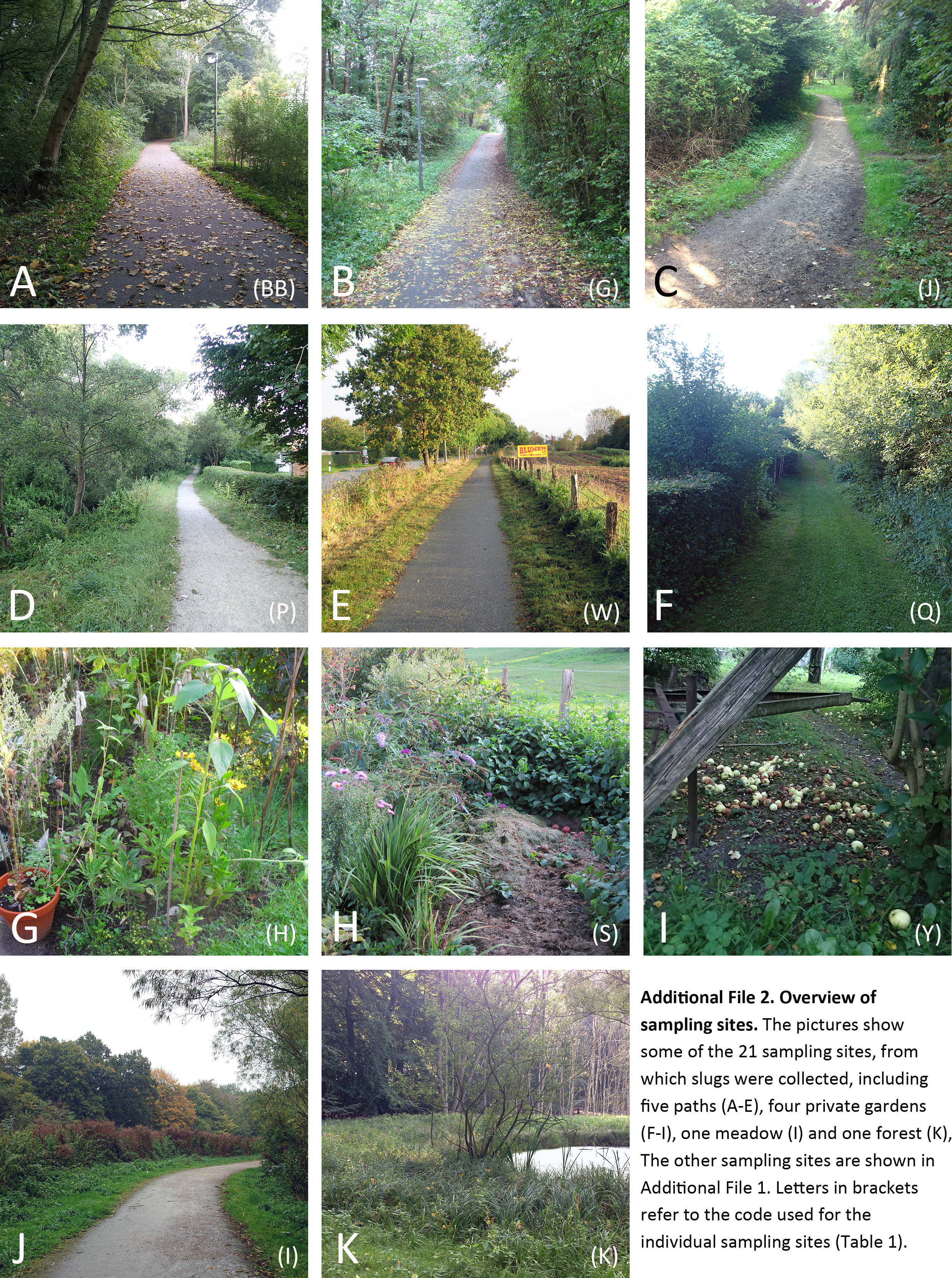

Supplement: Additional file 2: — Overview of sampling sites. The pictures show some of the 21 sampling sites, from which slugs were collected, including five paths (A-E), four private gardens (F-I), one meadow (J) and one forest (K). The other sampling sites are shown in Additional file 2. Letters in brackets refer to the code used for the individual sampling sites (Table 1). [file 12898_2015_50_MOESM2_ESM.jpg]

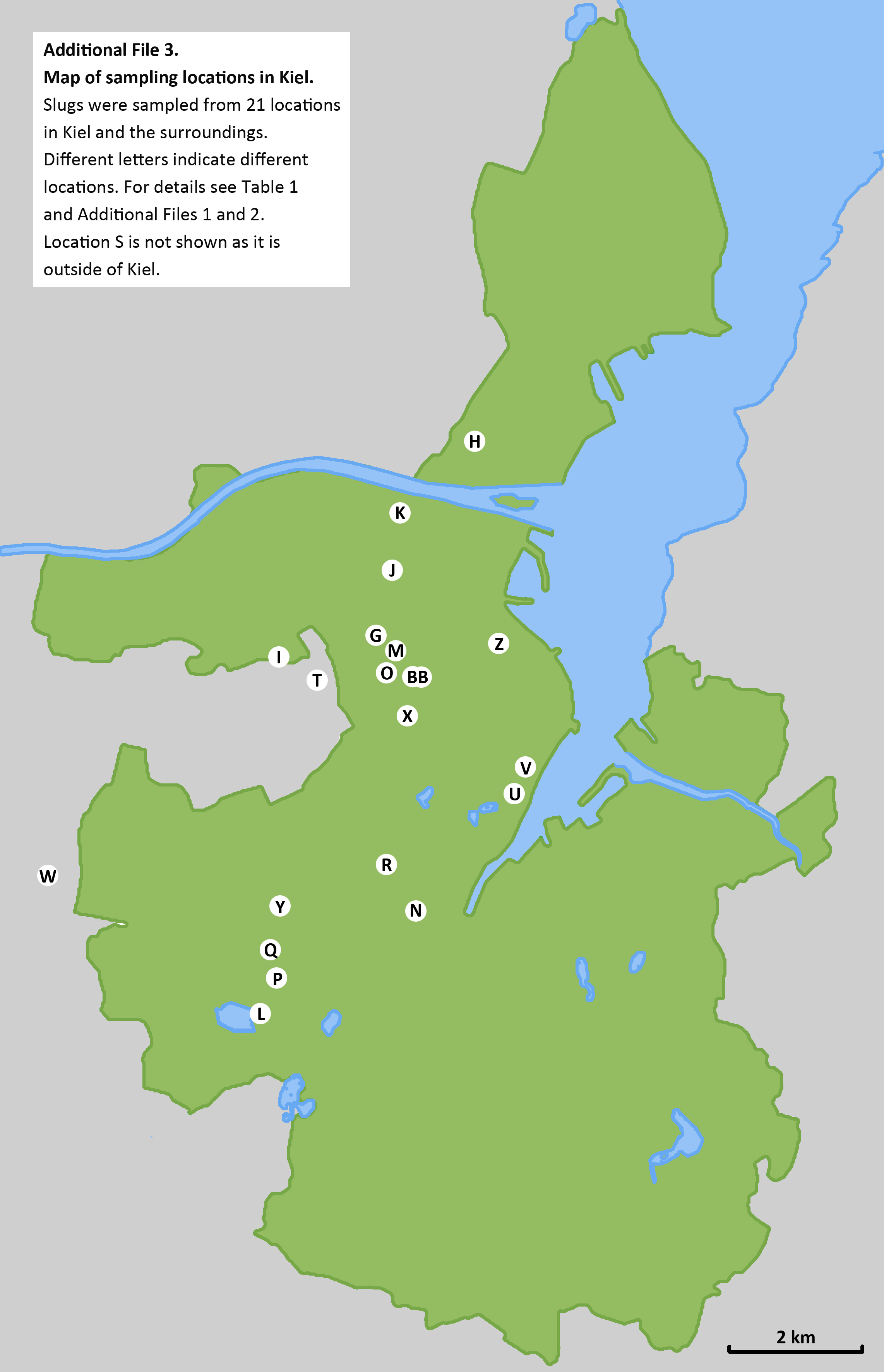

Supplement: Additional file 3: — Map of sampling locations in Kiel. Slugs were sampled from 21 locations in Kiel and the surroundings. Different letters indicate different locations. For details see Table 1 and Additional Files 2 and 3. Location S is not shown as it is outside of Kiel. [file 12898_2015_50_MOESM3_ESM.jpg]
